# Ginkgo Biloba Extract Ameliorates Age-Related Mitochondrial Deficits in Human iPSCs and Their Derived Neurons and Astrocytes

**DOI:** 10.3390/antiox15060689

**Published:** 2026-05-29

**Authors:** Imane Lejri, Amandine Grimm, Anne Eckert

**Affiliations:** 1 Neurobiology Lab for Brain Aging and Mental Health, University Psychiatric Clinics UPKl, 4002 Basel, Switzerland; imane.lejri@upk.ch (I.L.); amandine.grimm@upk.ch (A.G.); 2 Department of Biomedicine, University of Basel, 4031 Basel, Switzerland

**Keywords:** aging, mitochondrial dysfunction, induced pluripotent stem cells (iPSCs), iPSC-derived neurons, iPSC-derived astrocytes, Ginkgo biloba extract, bioenergetics, oxidative stress

## Abstract

Mitochondrial dysfunction is a central feature of aging, driving bioenergetic decline, increased oxidative stress, and increased vulnerability to neurodegenerative diseases. Human induced pluripotent stem cells (iPSCs) and iPSC-derived neurons provide powerful models to study these processes. Ginkgo biloba extract GBE LI1370 (GBE) has demonstrated antioxidant and mitochondria-protective properties in preclinical models, including improvements in mitochondrial membrane potential, reduction in reactive oxygen species, and enhanced neuronal survival. However, its effects on mitochondrial function in human iPSCs and their differentiated derivatives in the context of aging have not yet been investigated. This study evaluated the mitochondrial protective effects of GBE (100 µg/mL) in an established iPSC-based model of aging and in neurons and astrocytes derived from aged iPSCs. Mitochondrial parameters, including ATP production, mitochondrial membrane potential (MMP), mitochondrial reactive oxygen species (mtROS), superoxide levels, and mitochondrial respiration, were assessed. Aged iPSCs exhibited reduced ATP production and MMP, together with increased mtROS and superoxide levels compared to young controls. Astrocytes derived from aged iPSCs also displayed mitochondrial dysfunction. Treatment with GBE for 24 h increased ATP production and MMP, reduced oxidative stress, and improved mitochondrial respiration in both young and aged iPSCs, as well as in aged iPSC-derived neurons and astrocytes. These preliminary donor-based findings support further investigation of GBE-associated mitochondrial responses in human donor-derived cellular models of aging and warrant validation in larger donor cohorts.

## 1. Introduction

Mitochondrial dysfunction is a hallmark of aging and a key contributor to cellular decline and neurodegeneration [1,2,3,4,5]. The aging process is closely associated with oxidative stress, contributing to mitochondrial dysfunction and cellular damage [3,6,7]. Age-related alterations in mitochondrial function include reduced ATP production, impaired mitochondrial membrane potential (MMP), and elevated mitochondrial reactive oxygen species (mtROS), leading to oxidative stress and bioenergetic failure [3,8,9].

Among the various pharmacological properties of Ginkgo biloba extract (GBE), its antioxidant potential, anti-apoptotic effects, and especially its role in mitochondrial protection have been widely documented [6,10,11,12,13]. In the context of brain aging, GBE has been shown to preserve mitochondrial respiratory chain complexes I, IV, and V, improve mitochondrial membrane potential and morphology, and reduce mitochondrial ROS levels. For instance, GBE protects against mitochondrial dysfunction in platelets from both young and old mice; however, in the hippocampus, protective effects were observed only in old mice, potentially due to age-related increases in blood–brain barrier permeability [14]. GBE has broad applications in age-related conditions, including Alzheimer’s disease. For example, EGb 761, a standardized GBE formulation, demonstrated neuroprotective effects in a cellular model of Alzheimer’s disease using PC12 cells expressing an amyloid precursor protein mutation [15]. Previously, GBE (24 h, 100 µg/mL) has been shown to improve mitochondrial function and energy metabolism in SH-SY5Y neuroblastoma cells, enhancing mitochondrial coupling efficiency and promoting electron transport chain (ETC) activity [16]. Further, GBE treatment demonstrated the capacity to modulate mitochondrial complexes I, III, and IV, increase mitochondrial DNA (mtDNA) content, and promote neuronal plasticity by stimulating neurite outgrowth and activating the PI3K/Akt/mTOR signaling pathway [12]. In addition, recent findings demonstrated that proanthocyanidins (PACs), key active constituents of GBE, improve mitochondrial bioenergetics and stimulate neuronal outgrowth in vitro, reinforcing the mitochondria-targeted and neurotrophic actions of GBE and its components under physiological conditions [13].

Human induced pluripotent stem cells (iPSCs) and their differentiated derivatives, such as cortical neurons, represent valuable models to study the cellular impact of aging in a human genetic background [8,17,18,19,20]. More recently, compelling evidence highlighted that iPSCs from aged donors retain mitochondrial aging signatures [9]. Aged iPSCs display impaired mitochondrial bioenergetics, reduced ATP production, diminished mitochondrial membrane potential, and increased mitochondrial ROS levels, highlighting persistent mitochondrial dysfunction at the cellular level [9]. Strikingly, similar data were obtained in aged iPSC-derived neurons [8,21]. In contrast, iPSCs and neurons derived from young donors exhibit preserved mitochondrial function, highlighting an age-dependent divergence in bioenergetic and redox status [8]. These findings suggest that iPSCs and iPSC-derived neurons from aged individuals are a valuable model for screening mitochondria-targeted interventions aimed at promoting healthy brain aging.

Astrocytes play a crucial role in maintaining neuronal homeostasis, providing metabolic and trophic support, regulating neurotransmitter balance, and contributing to antioxidant defense [22]. In the aging and neurodegenerative brain, astrocytic dysfunction profoundly affects neuronal survival and mitochondrial health [23,24]. Therefore, assessing the effect of GBE on both iPSC-derived neurons and astrocytes is essential to capture the cell-type-specific responses and the intercellular metabolic coupling that occurs in the CNS. While neurons are highly energy-demanding and directly affected by mitochondrial dysfunction, astrocytes are key regulators of the neuronal energy supply through lactate shuttling and mitochondrial transfer [25]. Studying both cell types allows evaluation of whether GBE affects mitochondrial and metabolic phenotypes in neurons, astrocytes, or both, rather than addressing specific intercellular mechanisms. Moreover, comparing iPSC-derived astrocytes and neurons from aged donors enables the evaluation of how cellular aging may influence responsiveness to GBE in distinct but interdependent neural populations [24,26]. Despite extensive documentation of GBE’s biological activities in models of neurodegeneration and aging, no study has directly compared its effects on mitochondrial function in young versus aged human iPSCs. Furthermore, its potential antioxidant and bioenergetic effects have not been investigated in neurons and astrocytes derived from aged iPSCs. To address this gap, we compared the effects of GBE in young and aged donor iPSCs and conducted key experiments in neurons and astrocytes derived exclusively from aged iPSCs to specifically assess antioxidant effects in the context of aging. This approach allowed us to evaluate age-dependent differences in GBE responsiveness at the iPSC level while assessing the relevance of GBE treatment in aged donor-derived neurons and astrocytes. Given the limited donor cohort, this study was designed as an exploratory proof-of-concept investigation to assess whether age-associated mitochondrial phenotypes and acute GBE responsiveness could be detected in a human donor-derived cellular model.

## 2. Materials and Methods

### 2.1. Chemicals and Reagents

The ATPlite™ 1-step luminescence assay was purchased from PerkinElmer (Waltham, MA, USA). Dimethyl sulfoxide (DMSO) Hybri-Max, Tetramethylrhodamine methyl ester perchlorate (TMRM), Hanks’ Balanced Salt Solution (HBSS), and Dihydrorhodamine 123 (DHR) were obtained from Sigma-Aldrich (St. Louis, MO, USA). MitoSOX™ Red Mitochondrial Superoxide Indicator was purchased from Invitrogen (Waltham, MA, USA). The Seahorse XFp Cell Mito Stress Test Kit, Seahorse XF Calibrant Solution, and Seahorse XF DMEM Assay Medium (pH 7.4), as well as glucose, pyruvate, and glutamine, were obtained from Agilent Technologies (Santa Clara, CA, USA). The Cellartis DEF-CS 500 Culture System, Cellartis DEF-CS 500 COAT-1, RHB-A, RHB-BASAL, and NDiff N2 were supplied by Takara Bio (Kusatsu, Shiga, Japan). Phosphate-Buffered Saline (PBS) was from Dominique DUTSCHER SAS (Bernolsheim, France). Advanced DMEM/F-12, B-27 Supplement (50X), distilled water, Geltrex™ LDEV-Free hESC-Qualified Reduced Growth Factor Basement Membrane Matrix, GlutaMAX™, Neurobasal™ Medium minus phenol red, PSC Neural Induction Medium, StemPro™ Accutase™ Cell Dissociation Reagent, and TrypLE™ Select Enzyme were all acquired from Gibco (Waltham, MA, USA). Recombinant human EGF, Recombinant Human FGF-basic, Recombinant Human/Murine/Rat BDNF, and Recombinant Human BMP-4 protein were obtained from PeproTech (Cranbury, NJ, USA). The ROCK inhibitor Y-27632 was purchased from Selleck Chemicals (Houston, TX, USA). The standardized Ginkgo biloba extract (GBE, LI1370; article number: 111279, lot number: 99151) was produced and supplied by OM Pharma, Switzerland. According to the Pharmacopoea Europaea, the standardized extract contains 22.0–27.0% ginkgo flavone glycosides and 5.4–6.6% terpenoids.

### 2.2. Experimental Design

The concentration of 100 µg/mL was selected as a biologically active condition based on prior in vitro dose-ranging studies from our group and others showing efficacy within the 1–100 µg/mL range, with 100 µg/mL consistently producing beneficial mitochondrial effects without evidence of overt cytotoxicity [12,13,16]. The present study was therefore designed as a proof-of-concept evaluation of a preselected active condition rather than a full pharmacological dose–response characterization. A 24 h exposure period was selected to capture early mitochondrial and redox responses prior to longer-term adaptive changes and based on prior experimental observations showing measurable mitochondrial responses within this time frame. The effects of GBE at a concentration of 100 µg/mL were assessed after 24 h of treatment in human induced pluripotent stem cells (iPSCs). The following parameters were quantified: ATP production, cell viability, mitochondrial membrane potential (MMP), reactive oxygen species (ROS) levels, and mitochondrial respiration.

In addition, key experiments were performed in iPSC-derived neurons and astrocytes from aged donors, focusing on the most relevant mitochondrial readouts associated with aging-related dysfunction. Specifically, ATP production, MMP, mitochondrial ROS emission (DHR fluorescence), and mitochondrial superoxide anion levels (MitoSOX fluorescence) were measured to extend the observations obtained in iPSCs and to assess whether GBE exposure was associated with modulation of mitochondrial parameters in differentiated neuronal and astrocytic cells derived from aged donors. To ensure consistent experimental conditions, the culture medium was refreshed daily for all groups. Cells were either treated with GBE (100 µg/mL) or maintained in fresh medium alone as an untreated control.

### 2.3. Cell Culture

#### 2.3.1. Human iPSCs and Neuron-Derived Cell Culture Model

Human iPSC lines were obtained from Takara Bio and the Coriell Institute (Table 1). The study was conducted using a selection of extensively characterized iPSCs from young (*n* = 3, ages 24–32 years) and aged donors (*n* = 3, ages 62–72 years), as well as the neuronal derivatives from aged donors. This design allowed us to compare age-related differences in mitochondrial function and oxidative stress and to evaluate the effects of GBE in both young and aged cellular contexts.

#### 2.3.2. iPSC Maintenance and Neuronal Differentiation

Human iPSC cells were maintained under feeder-free conditions on Cellartis DEF-CS COAT-1-coated plates, using Cellartis DEF-CS basal medium supplemented with GF-1 (1:333) and GF-2 (1:1000), proprietary supplements provided as part of the Cellartis culture system that support pluripotent stem cell maintenance according to the manufacturer’s protocol. To minimize cell death after passaging or plating, GF-3 (1:1000), a transient proprietary supplement included during seeding or replating, was added to the medium and removed with medium exchange the following day according to the manufacturer’s recommendations. Cells were passaged once a week using TrypLE™ Select and cultured in a humidified incubator at 37 °C with 5% CO_2_. The medium was refreshed daily.

Neural induction and subsequent neuronal differentiation were performed exclusively on iPSC-derived neurons from aged donors, following the established protocol of our group [8,21]. iPSCs were first expanded and differentiated into neural stem cells (NSCs) after 7 days, followed by further differentiation into neurons after 28 days, using Geltrex-coated plates and stage-specific media. Representative brightfield images documenting morphological progression during neural induction from iPSCs to NSCs are provided in Supplementary Figure S1. Validation of neural stem cell identity derived from iPSCs from aged donors was performed by immunostaining for Nestin (green) and Sox2 (orange), with DAPI (blue) nuclear counterstaining, and confirmed robust neural stem cell marker expression in all three donor lines (GM25430, AG25269, and AG27602). Representative quality control images of neural stem cells derived from aged iPSCs by Nestin/Sox2 immunostaining are provided in Supplementary Figure S2. For the key experiments, neuronal differentiation was carried out exclusively on iPSCs derived from aged donors to specifically investigate aging-related mitochondrial alterations. Neuronal identity was validated by immunocytochemistry using MAP2 (microtubule-associated protein 2, green) as a neuronal marker and DAPI (blue) for nuclear staining. Representative quality control images of neuronal cells derived from aged iPSCs by MAP2 immunostaining are provided in Supplementary Figure S3.

#### 2.3.3. Differentiation of Aged NPCs into Astrocytes

For astrocyte differentiation, neural stem cells (NSCs) dissociated with StemPro Accutase were seeded onto Geltrex-coated 6-well plates at a density of 7.6 × 10^4^ cells per well or onto Geltrex-coated 10 cm^2^ dishes at a density of 4.5 × 10^5^ cells per dish in RHB-A medium, a defined medium supporting astrocyte differentiation and maintenance, containing 10 ng/mL BMP-4, a gliogenic factor promoting astrocytic lineage commitment and maturation. Geltrex coating was used to support cell attachment and reproducible differentiation conditions. To enhance survival after dissociation, 10 µM ROCK inhibitor Y-27632 was included overnight following seeding as a transient survival-support measure to prevent dissociation-induced cell death, consistent with standard stem cell culture practice. The medium was replaced the following day with ROCK inhibitor-free medium. Half of the culture medium was refreshed every other day for 22 days, keeping the BMP-4 concentration constant at 10 ng/mL throughout the differentiation process to support phenotypic stability. Differentiation toward an astrocytic phenotype was supported by sustained BMP-4 exposure and confirmed by immunostaining for multiple astrocytic markers, including GFAP (anti-GFAP antibody rabbit, Abcam, ab68428), GLAST (anti-EAAT1 antibody chicken, SYSY Synaptic System, 250 116), and S100B (anti-S100B antibody guinea pig, SYSY Synaptic System, 287 004), under the established differentiation conditions (Supplementary Figure S4A) [27]. Cells were maintained in the same culture plate throughout the 22-day differentiation period, with no intermediate replating or passaging. After the 22-day differentiation phase, astrocytes were maintained in RHB-A/BMP-4-containing maintenance medium until experimental use.

In addition, baseline characterization experiments comparing astrocytes derived from young and aged donors were performed to establish age-associated mitochondrial and oxidative deficits in the astrocyte model (Supplementary Figures S5C and S6). These characterization experiments were conducted to validate the aged astrocyte phenotype prior to the GBE intervention studies. GBE treatment experiments were performed exclusively in aged donor-derived astrocytes.

#### 2.3.4. Post-Differentiation Replating of iPSC-Derived Astrocytes for Experiments

For experimental applications, aged iPSC-derived astrocytes were replated into the appropriate assay formats to enhance cell viability and ensure reproducibility under small-volume culture conditions. Replating was performed only after completion of astrocyte differentiation for experimental assays. Fully differentiated astrocytes were detached using StemPro Accutase supplemented with 10 µM ROCK inhibitor Y-27632 (45 min, 37 °C) and subsequently seeded into Geltrex-coated 96-well plates at a density of 6.0 × 10^5^ cells per well in RHB-A medium containing BMP-4.

To minimize cell loss following replating, 10 µM ROCK inhibitor Y-27632 was maintained overnight only, after which the medium was replaced with ROCK inhibitor-free medium the next day. Half of the culture medium was refreshed every other day for 7 days prior to initiating treatment with GBE. Maintenance of differentiated astrocytic properties after replating was confirmed by astrocytic marker immunostaining quality control, including GFAP, GLAST, and S100B, performed under the same experimental culture conditions. Representative quality control images of astrocytes replated with or without Geltrex coating are provided in Supplementary Figure S4B, illustrating improved attachment under coated conditions.

### 2.4. ATP Level Measurements

The total cellular ATP content was measured using a bioluminescence assay (ViaLight™ HT, Cambrex Bio Science, Walkersville, MD, USA), following the manufacturer’s instructions and as previously described [9]. Cells were seeded into white 96-well plates (6.0  ×  10^5^ cells per well). For iPSCs, Cellartis DEF-CS COAT-1-coated plates were used, while for iPSC-derived neurons and astrocytes, Geltrex-coated plates were employed. The assay is based on the luciferase-catalyzed reaction between ATP and luciferin, generating luminescence proportional to the ATP concentration. The emitted light was detected using a Cytation 3 Cell Imaging Multi-Mode Plate Reader (BioTek, Agilent, Switzerland).

### 2.5. Mitochondrial Membrane Potential (MMP) Detection

The mitochondrial membrane potential (MMP) was assessed using tetramethylrhodamine methyl ester perchlorate (TMRM), a fluorescent dye sensitive to membrane potential changes. Cells were seeded into black 96-well cell culture plates (6.0  ×  10^5^ cells per well). After plating, cells were incubated with TMRM (0.4 µM) for 30 min at 37 °C [21]. Subsequently, cells were washed twice with Hank’s Balanced Salt Solution (HBSS) to remove excess dye. Fluorescence intensity was measured using a Cytation3 Cell Imaging Multi-Mode Plate Reader (BioTek) at 531 nm excitation and 595 nm emission. The transmembrane distribution of TMRM is directly dependent on the mitochondrial membrane potential, with higher fluorescence indicating more polarized mitochondria. TMRM fluorescence values were normalized to CellTracker Blue fluorescence to account for differences in viable cell coverage.

### 2.6. Reactive Oxygen Species (ROS) Detection

Mitochondrial reactive oxygen species (mtROS) and mitochondrial superoxide anion levels were quantified using dihydrorhodamine 123 (DHR) and MitoSOX™ Red Mitochondrial Superoxide Indicator, respectively. Cells were plated in black 96-well plates, coated with Cellartis DEF-CS COAT-1 for iPSCs and Geltrex for iPSC-derived neurons and astrocytes (6.0  ×  10^5^ cells per well). Following GBE treatment, cells were incubated with 5 µM MitoSOX for 2 h at room temperature, based on previously established assay conditions used in our prior studies with human iPSC-derived neurons [21], which provided robust and reproducible detection of mitochondrial superoxide. MitoSOX incubation conditions may vary depending on cell type and assay platform and may require empirical optimization across experimental systems. After incubation, cells were washed twice with HBSS (Sigma). DHR, upon oxidation, is converted to cationic rhodamine 123, which accumulates in mitochondria and emits green fluorescence detected at 485 nm excitation/538 nm emission. MitoSOX, specifically oxidized by mitochondrial superoxide, emits red fluorescence detected at 535 nm excitation/595 nm emission. Fluorescence intensity was measured using a Cytation3 Cell Imaging Multi-Mode Reader (BioTek). The signal is proportional to the levels of mtROS or mitochondrial superoxide anion radicals. Importantly, fluorescence measurements were performed directly in intact adherent cells without cell lysis or transfer of supernatants. CellTracker Blue was not present during DHR or MitoSOX acquisition. Following completion of the ROS fluorescence measurements, cells were washed and subsequently incubated with CellTracker Blue in the same wells for normalization purposes using a separate fluorescence acquisition step. DHR and MitoSOX fluorescence values were normalized to CellTracker Blue fluorescence to account for differences in viable cell coverage.

### 2.7. Determination of Oxygen Consumption Rate (OCR) and Extracellular Acidification Rate (ECAR)

Mitochondrial respiration (OCR) and glycolysis (ECAR) were simultaneously measured in young and aged iSPCs cells in real time using the Seahorse XF HS Mini Analyzer (Agilent, Switzerland) [8]. XF cell culture microplates (Agilent Seahorse Bioscience) were pre-coated with Cellartis DEF-CS COAT-1. iPSC cells were seeded at 3.0  ×  10^5^ cells per well in 80 µL of medium and incubated overnight. After attachment, cells were treated for 24 h with the respective experimental conditions. Following treatment, cells were washed with assay buffer and incubated with 200 µL of Seahorse XF assay medium at 37 °C in a CO_2_-free incubator for 1 h. After equilibration, the medium was replaced with 160 µL of fresh assay medium before starting the measurement. The assay medium consisted of Seahorse XF DMEM (pH 7.4) supplemented with 18 mM glucose, 2 mM L-glutamine, and 4 mM pyruvate. OCR and ECAR were recorded for 30 min in the XF Analyzer. Data analysis was performed using the Agilent Seahorse Analytics platform, which automatically calculated key bioenergetic parameters including basal respiration, spare respiratory capacity, maximal respiration, proton leak, ATP-linked respiration, and non-mitochondrial respiration.

### 2.8. Data Normalization

Data were normalized to the viable cell area assessed using CellTracker Blue (CTB) staining [28]. CTB fluorescence was used as an indicator of cell number/cell content to normalize all quantitative outputs, thereby adjusting for differences in viable cell coverage. Following incubation with CTB (5 µM, 30 min, 37 °C, dark) and HBSS washing, fluorescence was measured at 353/466 nm using a Cytation 3 plate reader (Agilent). For ATP measurements, CTB staining and fluorescence measurement were performed prior to cell lysis, after which ATP luminescence was quantified. Under our assay conditions, prior CTB staining did not interfere with the ATP luminescence readout. In all other fluorescence-based assays, including TMRM, DHR, and MitoSOX measurements, CTB staining was performed only after completion of the primary fluorescence readout. Fluorescence acquisition for CTB and the respective experimental probes was therefore performed sequentially with separate excitation/emission settings to minimize potential spectral interference between fluorophores. No cell lysis was performed for TMRM, DHR, or MitoSOX assays. No protein-based normalization was used in these fluorimetric assays.

### 2.9. Statistical Analysis

**Figure 1:**
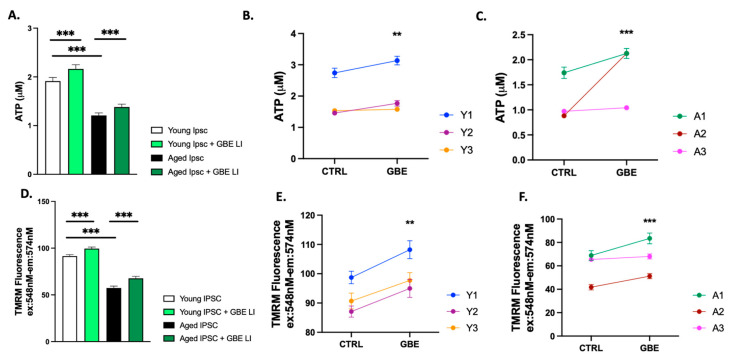
Effect of a 24 h treatment with GBE (100 µg/mL) on bioenergetics in human iPSCs from young and aged donors compared to the CTRL condition. Relative ATP concentration (**A**–**C**) and mitochondrial membrane potential (MMP) levels (**D**–**F**) were measured in young and aged donor-derived iPSCs. Bar graphs represent mean ± SEM from seven independent experiments using three human iPSC lines from young donors and three from aged donors (ATP: 36–46 technical replicates per donor; MMP: 41–45 technical replicates per donor; biological *n* = 3 donors per age group). Donor-specific responses for ATP levels in young (**B**) and aged (**C**) iPSC lines following GBE treatment. Donor-specific responses for MMP levels in young (**E**) and aged (**F**) iPSC lines following GBE treatment. Y1–Y3 correspond to independent young donor-derived iPSC lines and A1–A3 correspond to independent aged donor-derived iPSC lines. These plots show the response of each individual biological replicate (donor line) to GBE treatment and were included to illustrate the consistency and reproducibility of the GBE effect across independent donor-derived cell lines, rather than relying solely on pooled mean responses. For ATP (**A**), two-way ANOVA revealed a significant effect of aging (young vs. aged iPSCs, *p* = 0.006) and treatment (GBE vs. CTRL, *p* < 0.001). For MMP (**D**), two-way ANOVA showed significant effects of aging (*p* < 0.001) and treatment (*p* < 0.001). At the donor level (**B**,**C**,**E**,**F**), two-way ANOVA with factor treatment confirmed a consistent main effect of GBE across individual donor lines. Post hoc multiple comparisons were performed using Tukey’s test; ** *p* < 0.01, *** *p* < 0.001.

**Figure 2:**
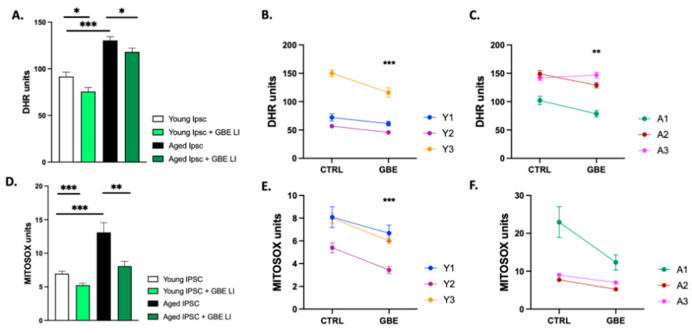
Effect of a 24 h treatment with GBE (100 µg/mL) on ROS production in human iPSCs from young and aged donors compared to the CTRL condition. Mitochondrial ROS emission measured by DHR fluorescence (**A**–**C**) and mitochondrial superoxide levels measured by MitoSOX fluorescence (**D**–**F**) were assessed in young and aged donor-derived iPSCs. Bar graphs represent mean ± SEM from seven independent experiments using three human iPSC lines from young donors and three from aged donors (DHR: 39–50 technical replicates per donor; MitoSOX: 40–45 technical replicates per donor; biological *n* = 3 donors per age group). Donor-specific responses for DHR fluorescence in young (**B**) and aged (**C**) iPSC lines following GBE treatment. Donor-specific responses for MitoSOX fluorescence in young (**E**) and aged (**F**) iPSC lines following GBE treatment. Y1–Y3 correspond to independent young donor-derived iPSC lines and A1–A3 correspond to independent aged donor-derived iPSC lines. These plots show the response of each individual biological replicate (donor line) to GBE treatment and were included to illustrate the consistency and reproducibility of the GBE effect across independent donor-derived cell lines, rather than relying solely on pooled mean responses. For DHR fluorescence (**A**), two-way ANOVA revealed significant effects of aging (young vs. aged iPSCs, *p* < 0.001) and treatment (GBE vs. CTRL, *p* < 0.001). For MitoSOX fluorescence (**D**), two-way ANOVA also showed significant effects of aging (*p* < 0.001) and treatment (*p* < 0.001). At the donor level (**B**,**C**,**E**,**F**), two-way ANOVA with factor treatment confirmed a consistent main effect of GBE across individual donor lines. Post hoc multiple comparisons were performed using Tukey’s test; * *p* < 0.05,** *p* < 0.01, *** *p* < 0.001.

Data are presented as mean ± SEM. Statistical analyses were performed using GraphPad Prism software (version 9.3.1; GraphPad Software, San Diego, CA, USA). For experiments involving two independent variables (age and treatment), statistical significance was assessed using a two-way analysis of variance (two-way ANOVA). When significant effects were detected, post hoc comparisons were performed using Tukey’s multiple comparisons test. For comparisons between two groups, an unpaired two-tailed Student’s *t*-test was applied. A *p*-value < 0.05 was considered statistically significant. Biological replicates were defined as donor lines (n), whereas repeated wells or measurements within each donor line were considered technical replicates used to improve assay robustness and measurement reliability. Technical replicates were not considered independent biological observations. Young donor-derived lines are indicated as Y1–Y3, whereas aged donor-derived lines are indicated as A1–A3 in donor-specific plots. Donor-specific responses to GBE treatment are additionally study Figure 1, Figure 2 to illustrate consistency across biological replicates.

## 3. Results

### 3.1. Age-Associated Mitochondrial Dysfunction in iPSCs and iPSC-Derived Neurons

We first confirm the age-associated differences in mitochondrial function that we have highlighted in previous studies [9,21]. Key mitochondrial parameters were quantified in both iPSCs and iPSC-derived neurons from young and aged donors, including ATP production, MMP, mtROS, and mSuperox. Data are summarized in the form of radar plots in Figure S5.

In iPSCs, cells from aged donors exhibited reduced ATP production and decreased MMP compared to those from young donors (Figure S5A). In parallel, aged iPSCs showed elevated levels of both mtROS and mSuperox, indicating increased oxidative stress and potential mitochondrial dysfunction (Figure S5A). In iPSC-derived neurons, similar age-related alterations were observed. Neurons derived from aged donors displayed lower ATP production and MMP, alongside increased mtROS and mSuperoxide levels compared to neurons from young donors (Figure S5B).

Having identified a distinct aging-related mitochondrial dysfunction profile marked by impaired ATP production, reduced mitochondrial membrane potential, and elevated oxidative stress, we next sought to determine whether GBE exposure was associated with modulation of these mitochondrial parameters. Specifically, we evaluated the capacity of GBE to modulate mitochondrial function in aged iPSCs and their derived neurons to assess whether GBE exposure was associated with changes in age-related mitochondrial parameters.

### 3.2. Astrocytes Derived from Aged Donors Display Signs of Mitochondrial Dysfunction

Before assessing GBE effects in aged astrocytes, we first characterized baseline mitochondrial function in astrocytes derived from young and aged donors.

To determine whether aging affects astrocytic bioenergetics and oxidative homeostasis, iPSC-derived astrocytes from young and aged donors were analyzed. Quantitative analysis revealed a clear decline in mitochondrial performance in astrocytes from aged donors compared with those from young controls (Figures S5C and S6).

Aged astrocytes exhibited a significant reduction in ATP production and MMP levels (ATP: 2.4 vs. 1.9 µM; MMP: 162.4 vs. 103.1 fluorescence units; Figure S6A,B), accompanied by a marked increase in mtROS and superoxide anion level generation (DHR fluorescence: 30.9 vs. 50.1 fluorescence units; MitoSOX fluorescence: 9.5 vs. 10.9 fluorescence units; Figure S6C,D).

Together, these findings indicate that astrocytes from aged donors exhibit marked mitochondrial impairment, reflected by diminished bioenergetic capacity and increased oxidative burden. To our knowledge, this study provides the first evidence of such age-associated mitochondrial alterations in human iPSC-derived astrocytes and establishes the aged astrocyte phenotype used for the subsequent GBE intervention experiments.

### 3.3. GBE Improves Mitochondrial Bioenergetics in Young and Aged iPSCs

To assess the impact of GBE on mitochondrial bioenergetics, human iPSCs from young and aged donors were exposed to 100 µg/mL GBE for 24 h.

At baseline, aged iPSCs exhibited significantly reduced ATP production and MMP levels compared to young iPSCs (ATP: 1.91 vs. 1.21 µM; MMP: 91.5 vs. 57.4 fluorescence units; Figure 1A,D). Treatment with GBE significantly increased ATP levels in both young and aged iPSCs (2.16 and 1.38 µM, respectively; Figure 1A), with a more pronounced response observed in young donor-derived cells. Similarly, MMP was enhanced by GBE in both donor groups, consistent with improved mitochondrial function, although the effect in aged iPSCs was more modest (99.5 and 67.7 fluorescence units, respectively; Figure 1D). Donor-specific analyses (Figure 1B,C,E,F) further showed that these responses were reproducible across independent donor-derived lines, with biological replicates exhibiting a consistent amelioration of response to GBE treatment for ATP and MMP.

Overall, these findings indicate that GBE promotes mitochondrial bioenergetic parameters in young iPSCs and exerts a more moderate beneficial effect in aged iPSCs.

### 3.4. GBE Reduces Oxidative Stress in Young and Aged iPSCs

With respect to oxidative stress, aged iPSCs displayed significantly elevated mitochondrial ROS and superoxide anion levels compared to young iPSCs (DHR fluorescence: 91.6 vs. 130.4 fluorescence units; MitoSOX fluorescence: 7 vs. 13.1fluorescence units; Figure 2A,D). GBE treatment markedly reduced mitochondrial ROS emission in both young and aged iPSCs, reaching 75.7 and 118.1 fluorescence units, respectively. A similar effect was observed for mitochondrial superoxide anions, with values decreasing to 5.3 in young and 8.1 in aged iPSCs (Figure 2A,D). Donor-specific analyses (Figure 2B,C,E,F) further showed that these responses were reproducible across independent donor-derived lines, with all biological replicates exhibiting a consistent direction of response to GBE treatment for DHR and MitoSOX measurements.

Thus, GBE reduces mitochondrial ROS and superoxide levels, consistent with antioxidant effects in both young and aged iPSCs.

Collectively, these results show that 24 h GBE treatment improves mitochondrial bioenergetic parameters and reduces oxidative stress in both young and aged iPSCs, supporting a potential role in mitigating age-associated mitochondrial dysfunction.

### 3.5. GBE Modulates Mitochondrial Respiratory and Glycolytic Bioenergetic Parameters in Aged iPSCs

To further assess the impact of GBE on cellular bioenergetics during aging, we performed a Seahorse XF Cell Mito Stress Test on iPSCs from young and aged donors.

**Figure 3:**
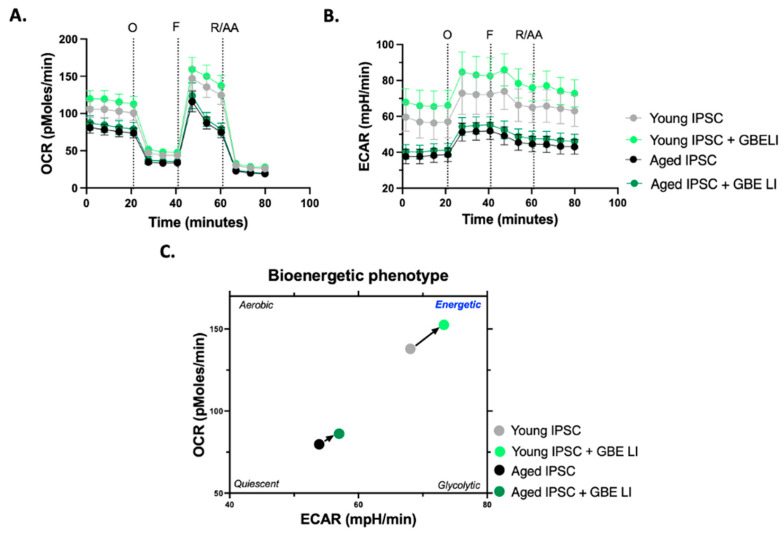
Effect of GBE (100 µg/mL) on mitochondrial respiration and glycolysis in young and aged iPSCs. (**A**) Oxygen consumption rate (OCR) and (**B**) extracellular acidification rate (ECAR) were measured in young and aged iPSCs using the Seahorse XF HS Mini Analyzer before and after sequential injection of mitochondrial stress test modulators. (**C**) Bioenergetic phenotype profile of young and aged iPSCs, displayed as mean basal ECAR (*x*-axis) versus mean basal OCR (*y*-axis). The black arrows indicate GBE-associated shifts in OCR and ECAR distributions in both donor groups. Values represent mean ± SEM of four independent experiments (OCR: *n* = 15–17 replicates per condition; ECAR: *n* = 24–30 replicates per condition) performed with three human iPSC lines from young donors and three human iPSC lines from aged donors. OCR, oxygen consumption rate; ECAR, extracellular acidification rate; O, oligomycin; F, FCCP; R, rotenone; AA, antimycin A.

At baseline, aged iPSCs exhibited a lower oxygen consumption rate (OCR) compared to young iPSCs (Figure 3A). GBE treatment significantly increased OCR in both donor groups. Similarly, the extracellular acidification rate (ECAR)—an indicator associated with glycolytic activity—was reduced in aged iPSCs relative to young cells (Figure 3B). Following GBE exposure, ECAR was elevated in both young and aged iPSCs. The OCR/ECAR bioenergetic profile (Figure 3C) showed distinct basal differences between young and aged cells and indicated GBE-associated changes in both respiratory and glycolytic parameters, reflected by shifts in the OCR–ECAR distribution in both donor groups.

### 3.6. GBE Modulates Mitochondrial Respiratory Parameters in Young and Aged iPSCs

**Figure 4:**
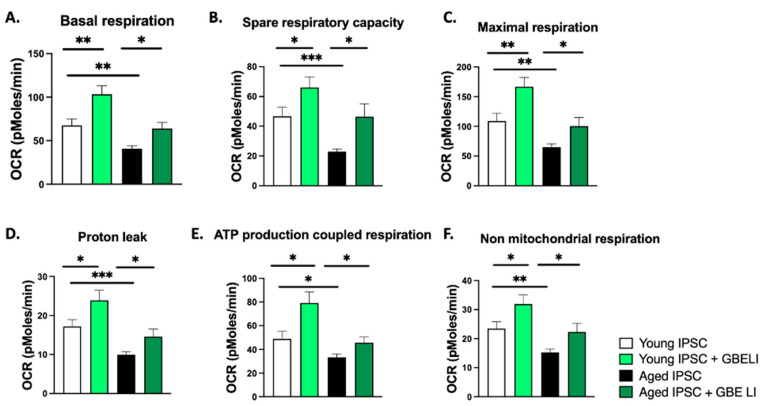
Effect of GBE (100 µg/mL) on mitochondrial respiratory parameters in young and aged iPSCs. OCR-based respiratory parameters, including (**A**) basal respiration, (**B**) spare respiratory capacity, (**C**) maximal respiration, (**D**) proton leak, (**E**) ATP production-linked respiration, and (**F**) non-mitochondrial respiration, were calculated using the Agilent Seahorse Analytics platform. Values represent mean ± SEM of four independent experiments (*n* = 14–21 replicates per group) performed with three human iPSC lines from young donors and three from aged donors. For (**A**,**C**): two-way ANOVA, aging effect (young vs. aged iPSCs) *p* < 0.001, treatment effect (GBE vs. CTRL) *p* < 0.001. For (**B**): two-way ANOVA, aging effect (young vs. aged iPSCs) *p* = 0.003, treatment effect (GBE vs. CTRL) *p* = 0.003. For (**D**): two-way ANOVA, aging effect (young vs. aged iPSCs) *p* = 0.004, treatment effect (GBE vs. CTRL) *p* < 0.001. For (**E**): two-way ANOVA, aging effect (young vs. aged iPSCs) *p* = 0.001, treatment effect (GBE vs. CTRL) *p* < 0.001. For (**F**): two-way ANOVA, aging effect (young vs. aged iPSCs) *p* = 0.003, treatment effect (GBE vs. CTRL) *p* < 0.001. Post hoc multiple comparisons were performed using Tukey’s test * *p* < 0.05, ** *p* < 0.01,*** *p* < 0.001.

Quantitative analysis of individual mitochondrial parameters (Figure 4) further characterized these effects. Aged iPSCs exhibited significantly reduced basal respiration (Figure 4A), spare respiratory capacity (Figure 4B), and maximal respiration (Figure 4C), proton leak (Figure 4D), ATP-linked respiration (Figure 4E), and non-mitochondrial respiration (Figure 4F) compared to young iPSCs. GBE was associated with increases in multiple respiratory parameters in both donor groups, although the magnitude and consistency of the response varied across parameters. Although young iPSCs displayed higher baseline respiration than aged iPSCs, GBE-associated responses were observed in both donor groups, with the magnitude of response varying across respiratory parameters. A descriptive normalization of GBE-induced changes (% change vs. respective control) across respiratory parameters is provided in Supplementary Figure S7 to facilitate comparison of relative treatment responses.

Overall, Seahorse analysis indicated that GBE modulated multiple mitochondrial respiratory parameters in both young and aged iPSCs, supporting a beneficial effect on cellular bioenergetics.

### 3.7. GBE Improves Mitochondrial Bioenergetic Parameters and Reduces Oxidative Stress in Aged iPSC-Derived Neurons and Astrocytes

**Figure 5:**
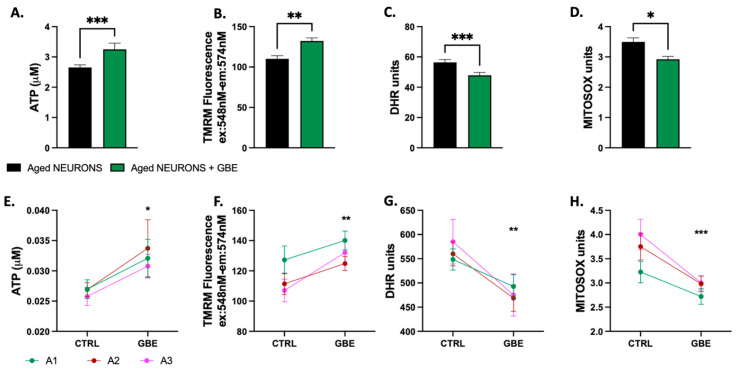
Effect of a 24 h treatment with GBE (100 µg/mL) on bioenergetics and oxidative stress in human iPSC-derived neurons from aged donors compared to the CTRL condition. (**A**–**D**) Relative ATP concentration (**A**), mitochondrial membrane potential (MMP) levels (**B**), intracellular oxidative stress measured by DHR fluorescence (**C**), and mitochondrial superoxide levels measured by MitoSOX fluorescence (**D**) were quantified in aged donor-derived neurons treated with medium alone (CTRL) or GBE. Bar graphs represent mean ± SEM from 5 to 7 independent experiments. For neurons: ATP, *n* = 40 technical replicates; MMP, *n* = 33 technical replicates; DHR, *n* = 36 technical replicates; MitoSOX, *n* = 30 technical replicates per condition (biological *n* = 3 donor lines). (**E**–**H**) Donor-specific responses of individual aged donor-derived neuronal lines (A1–A3) to GBE treatment for ATP (**E**), MMP (**F**), DHR fluorescence (**G**), and MitoSOX fluorescence (**H**). A1–A3 correspond to independent aged donor-derived neuronal lines. These plots show the response of each individual biological replicate (donor line) and were included to illustrate the consistency and reproducibility of the GBE effect across independent donor-derived neuronal lines, rather than relying solely on pooled mean responses. GBE treatment effects in pooled data (**A**–**D**) were analyzed using Student’s *t*-test. Donor-specific responses shown in (**E**–**H**) were analyzed by two-way ANOVA followed by Tukey’s multiple comparisons test. Significance is indicated as * *p* < 0.05, ** *p* < 0.01, *** *p* < 0.001.

**Figure 6:**
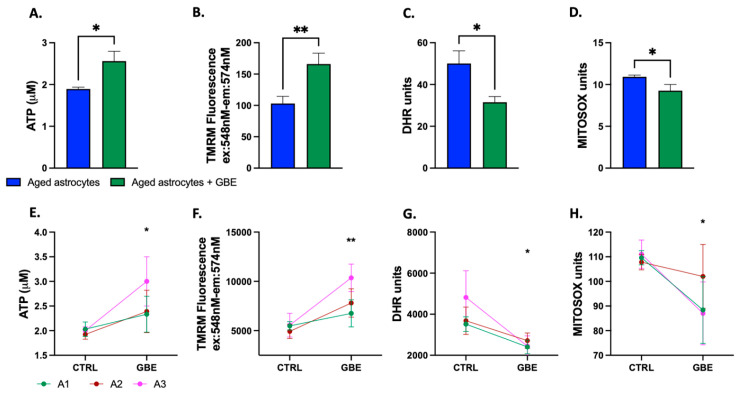
Effect of a 24 h treatment with GBE (100 µg/mL) on bioenergetics and oxidative stress in human iPSC-derived astrocytes from aged donors compared to the CTRL condition. (**A**–**D**) Relative ATP concentration (**A**), mitochondrial membrane potential (MMP) levels (**B**), intracellular oxidative stress measured by DHR fluorescence (**C**), and mitochondrial superoxide levels measured by MitoSOX fluorescence (**D**) were quantified in aged donor-derived astrocytes treated with medium alone (CTRL) or GBE. Bar graphs represent mean ± SEM from four independent experiments. For astrocytes: ATP, *n* = 11 technical replicates; MMP, *n* = 12 technical replicates; DHR, *n* = 12 technical replicates; MitoSOX, *n* = 14 technical replicates per condition (biological *n* = 3 donor lines). (**E**–**H**) Donor-specific responses of individual aged donor-derived astrocytic lines (A1–A3) to GBE treatment for ATP (**E**), MMP (**F**), DHR fluorescence (**G**), and MitoSOX fluorescence (**H**). A1–A3 correspond to independent aged donor-derived astrocytic lines. These plots show the response of each individual biological replicate (donor line) and were included to illustrate the consistency and reproducibility of the GBE effect across independent donor-derived astrocytic lines, rather than relying solely on pooled mean responses. GBE treatment effects in pooled data (**A**–**D**) were analyzed using Student’s *t*-test. Donor-specific responses shown in (**E**–**H**) were analyzed by two-way ANOVA followed by Tukey’s multiple comparisons test. Significance is indicated as * *p* < 0.05, ** *p* < 0.01.

Because neuronal and glial interactions critically determine brain bioenergetics and redox balance, assessing both iPSC-derived neurons and astrocytes provides a comprehensive view of mitochondrial function in the aged brain. Aged iPSC-derived neurons and astrocytes provide valuable in vitro models because of their availability and retention of aging-related mitochondrial impairments. To assess bioenergetics and redox homeostasis, we performed key experiments on iPSC-derived neurons and astrocytes from aged donors and examined the effects of a 24 h treatment with GBE (100 µg/mL) (Figure 5 and Figure 6).

GBE significantly increased ATP production and MMP levels compared to aged iPSC-derived neurons treated with the medium alone (ATP: 2.7 vs. 3.2 µM; MMP: 115.2 vs. 132.3 fluorescence units) (Figure 5A), consistent with improved mitochondrial function in aged neurons following GBE treatment. With respect to oxidative stress, GBE reduced mitochondrial ROS levels (Figure 5C) and decreased mitochondrial superoxide anion production (Figure 5D) (DHR fluorescence: 56.5 vs. 47.9 fluorescence units; MitoSOX fluorescence: 3.5 vs. 2.93 fluorescence units) in aged neurons. Donor-specific analyses (Figure 5E–H) further showed that these responses were reproducible across independent neuronal donor lines, with a consistent direction of response to GBE treatment across biological replicates.

Similarly, in aged iPSC-derived astrocytes, GBE treatment markedly improved mitochondrial function (Figure 6A–H). ATP production increased from 1.9 to 2.6 µM, and MMP values rose from 103.1 to 166.1 fluorescence units (Figure 5A,B). Moreover, GBE significantly decreased mitochondrial ROS levels (DHR fluorescence: 50.1 vs. 31.5 fluorescence units) and reduced mitochondrial superoxide anion production (MitoSOX fluorescence: 10.9 vs. 9.3 fluorescence units) (Figure 5G,H). Donor-specific analyses (Figure 6E–H) likewise demonstrated reproducible responses across independent astrocytic donor lines. A descriptive normalization of each parameter expressed as percentage change relative to its respective untreated control is provided in Supplementary Figure S8, allowing comparison of the relative magnitude and direction of GBE responses across cell types despite differences in absolute graph scales.

Taken together, these findings demonstrate that GBE enhances mitochondrial bioenergetic parameters and reduces oxidative stress in both aged iPSC-derived neurons and astrocytes.

### 3.8. Integrative Summary of GBE Effects

**Figure 7:**
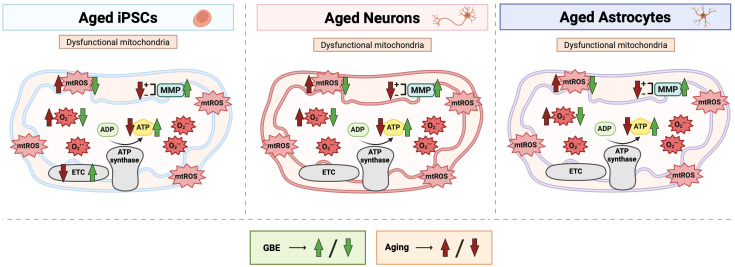
Summary of the effects of GBE on mitochondrial function in aged human iPSCs, neurons, and astrocytes. Schematic representation illustrating the major mitochondrial alterations observed during aging and the GBE-associated changes in mitochondrial parameters. Aged iPSCs, neurons, and astrocytes exhibit common features of mitochondrial dysfunction, including reduced mitochondrial membrane potential (MMP), impaired electron transport chain (ETC) activity, decreased ATP synthesis, and increased mitochondrial reactive oxygen species (mtROS) production. Treatment with GBE counteracts these aging-related deficits by enhancing ATP production, stabilizing MMP, supporting ETC function, and reducing mtROS accumulation. The bottom panel summarizes the direction of change for each parameter during aging (red arrows) and following GBE treatment (green arrows), illustrating the consistent bioenergetic improvement and reduction in oxidative stress across all three cell types. Created in BioRender. Grimm, A. (2026) https://BioRender.com/ifjthfh (accessed on 1 January 2026).

To summarize the overall effects of GBE, we generated an integrative schematic combining mitochondrial and metabolic readouts across all three cellular models (Figure 7).

Aged iPSCs, neurons, and astrocytes displayed characteristic features of mitochondrial dysfunction, including reduced ATP production and MMP levels, along with elevated mitochondrial ROS and superoxide levels. GBE treatment was associated with increases in ATP production and MMP, as well as reductions in mitochondrial oxidative stress markers across several cell types. In iPSCs, GBE was additionally associated with changes in mitochondrial respiratory and glycolytic bioenergetic parameters. Similar trends were observed in aged neurons and astrocytes, where GBE treatment was associated with stabilization of MMP and reduced mtROS accumulation. Collectively, Figure 7 summarizes the observed GBE-associated modulation of aging-related mitochondrial and bioenergetic parameters across pluripotent stem cells and their differentiated neuronal and astrocytic derivatives.

## 4. Discussion

Mitochondrial dysfunction is increasingly recognized as a central hallmark of cellular aging, contributing to progressive declines in energy metabolism, redox homeostasis, and regenerative capacity in both stem cells and differentiated tissues [4,29]. Our study provides new evidence that these mitochondrial impairments persist through cellular reprogramming and differentiation. We demonstrate that iPSCs, neurons, and astrocytes derived from aged donors exhibit reduced ATP production, lower MMP levels, and increased oxidative stress compared to those from young donors [8,9,30]. This finding confirms that donor age leaves a stable mitochondrial signature even after differentiation. Importantly, the detection of pronounced mitochondrial deficits in iPSC-derived astrocytes from aged donors compared to young donors highlights the significant contribution of glial aging to the overall metabolic deterioration of neural tissues [23,24,31,32].

Based on the literature, concentrations of Ginkgo biloba extracts or EGb 761^®^ used in in vitro studies typically range from 1 to 100 µg/mL, with beneficial biological effects reported within this interval [12,13,15,16]. A preselected biologically active concentration of 100 µg/mL was used based on previous in vitro studies reporting Ginkgo biloba extract concentrations ranging from 1 to 100 µg/mL, and on prior evidence demonstrating previously reported biological activities at 100 µg/mL [12,13,16]. Importantly, pharmacokinetic data indicate that circulating levels of Ginkgo biloba constituents in humans are typically in the low µg/mL range. However, circulating concentrations should be interpreted in the context of tissue distribution and repeated dosing. Standardized EGb 761 is clinically administered orally in humans, typically at doses of 120–240 mg/day, with documented pharmacological and clinical activity [33]. Constituents of EGb 761, including terpene trilactones such as bilobalide, have been reported to cross the blood–brain barrier and reach CNS tissue after oral administration, supporting the biological plausibility of central effects [34]. In addition, beneficial effects may involve not only direct CNS actions after blood–brain barrier penetration but also indirect peripheral and neurovascular mechanisms, including improved microcirculation and systemic antioxidant effects. Accordingly, the concentrations used here remain within a biologically relevant range commonly applied in cellular models. Although these conditions were chosen based on prior optimization studies, the present work does not establish a complete dose–response relationship nor define the temporal dynamics of GBE responsiveness in these human donor-derived systems. Future studies should systematically evaluate concentration dependence, repeated dosing paradigms, and longer treatment durations to determine whether the observed effects plateau, scale, or differ over time.

Crucially, this study also shows that GBE treatment was associated with improvements in several mitochondrial parameters in aged iPSCs and their derived neurons and astrocytes. A 24 h GBE treatment significantly increased ATP production and stabilized MMP while reducing mitochondrial ROS and superoxide levels. These effects indicate that GBE improves both mitochondrial efficiency and redox homeostasis. GBE induced improvements in aged iPSCs, neurons and astrocytes, which generally exhibited lower baseline mitochondrial function, although the magnitude of response varied across parameters and cell types. Thus, GBE was associated with improved mitochondrial function under aging conditions and may also support bioenergetic capacity in healthy cells, suggesting effects extending beyond aging-associated dysfunction. In addition to these effects, GBE treatment was associated with changes in mitochondrial respiratory and glycolytic bioenergetic parameters in aged iPSCs, suggesting modulation of cellular bioenergetics during the pluripotent stage. Donor-specific responses were analyzed separately and showed a consistent direction of GBE response across independent biological replicates, supporting the reproducibility of the observed effects despite limited donor numbers. However, the present data do not establish metabolic adaptability or energy reallocation mechanisms, which would require dedicated functional investigation.

The inclusion of astrocytes in this work further underscores the cellular breadth of GBE’s activity. Because mitochondrial bioenergetics and redox regulation are integral components of neuronal physiology, these parameters provide functional information at the cellular level, although they do not substitute for higher-order assays of synaptic or electrophysiological activity. Such improvements are consistent with the concept that glial mitochondrial health strongly influences neuronal function and contributes to brain aging [22,35]. The overall modulation of mitochondrial parameters observed following GBE treatment may reflect altered neuronal and glial bioenergetic states, ultimately promoting cellular homeostasis and stress resistance [8,36,37,38]. Compared with immortalized neuronal or astrocytic cell lines and rodent primary cultures, donor-derived human iPSC models offer the advantage of preserving human genetic background and aging-associated mitochondrial phenotypes that are often incompletely captured in simpler models. However, we acknowledge that these systems involve greater technical complexity, cost, and dependence on tightly controlled differentiation conditions, which may limit accessibility and reproducibility across laboratories. An additional limitation of this study is the restricted number of donor-derived iPSC lines and the exclusive use of male donor lines, which precludes assessment of sex-specific differences. Given the known influence of sex on mitochondrial function, aging trajectories, and cellular stress responses, future studies should include larger donor cohorts balanced for sex as a biological variable to evaluate the robustness and generalizability of these findings. Transient use of ROCK inhibitor was restricted to post-dissociation survival support and not maintained during differentiation, minimizing potential effects on cell identity or physiological properties. Furthermore, while mitochondrial and oxidative stress readouts were assessed using quantitative plate-reader fluorimetry, future studies combining these measurements with imaging-based approaches could provide complementary information regarding signal localization and biological interpretation.

Potential mechanisms proposed in previous studies suggest that GBE’s protective actions could involve both direct antioxidant effects and endogenous mitochondrial defense pathways, including NRF2 signaling [39]. Its bioactive constituents, flavonoids, terpene lactones, and proanthocyanidins, have previously been reported to preserve mitochondrial integrity and modulate apoptotic signaling [6,13,40]. Among them, bilobalide has been shown to inhibit ROS-induced apoptosis and block mitochondria-dependent caspase activation [14,41,42], while proanthocyanidins have been associated with improvements in mitochondrial morphology and neuronal plasticity [13]. In addition, previous studies suggest that GBE constituents may modulate intracellular and mitochondrial calcium homeostasis and attenuate calcium overload-associated mitochondrial dysfunction, which may also contribute to the beneficial effects observed here [10,11]. Although the present study demonstrates that GBE modulates mitochondrial and metabolic parameters in a human iPSC-derived neural aging model, the data remain primarily phenotypic and do not establish the molecular mechanisms underlying these effects. Pathways potentially involving NRF2 signaling, metabolic substrate utilization, or neuron–astrocyte coupling were not directly investigated and should therefore be considered hypotheses informed by previous literature rather than mechanisms demonstrated here. Thus, the present work should be regarded as an exploratory proof-of-concept study that provides a foundation for future mechanistic investigations.

Future studies could also explore whether GBE modulates lactate metabolism and mitochondrial transfer within neuron–astrocyte networks during aging. Given the crucial role of astrocyte-derived lactate in sustaining neuronal bioenergetics and synaptic function, and the emerging evidence for intercellular mitochondrial exchange as a compensatory mechanism under metabolic stress, assessing the influence of GBE on these processes could provide deeper insights into its system-level neuroprotective potential. Investigating how GBE affects mitochondrial trafficking, astrocyte-to-neuron metabolic coupling, and lactate shuttle efficiency will be critical to fully understand its capacity to restore energy homeostasis in the aged brain.

## 5. Conclusions

In summary, this study shows that GBE was associated with improvements in mitochondrial dysfunction in both young and aged human iPSCs and their derived neurons, improving ATP production, membrane potential, and oxidative stress markers, while modulating mitochondrial respiratory and glycolytic bioenergetic parameters. Although beneficial effects were observed in both donor groups, the magnitude of response varied across parameters and cell types, including beneficial responses observed in aged cells despite greater baseline mitochondrial impairments. Importantly, similar ameliorative effects were also observed in aged iPSC-derived astrocytes, where GBE enhanced mitochondrial bioenergetics and reduced oxidative stress, suggesting that these effects may extend beyond neurons to glial populations that are essential for maintaining neuronal energy supply and redox balance. Although these findings are preliminary and require validation in larger donor cohorts incorporating sex as a biological variable, they support further investigation of GBE-associated mitochondrial and oxidative stress responses in human donor-derived aging models, although validation in larger, mechanistically oriented, and sex-balanced studies remains necessary.

## Figures and Tables

**Table 1:** Human IPSC lines from young and aged donors were used in this study.

Identifier or Catalog Number	Sex	Age (Year)	Origin	Source	Donor Group
Cellartis^®^ Human iPS Cell Line 12	M	24	Human skin fibroblasts	Takara Bio	Young
Cellartis^®^ Human iPS Cell Line 18	M	32	Human skin fibroblasts	Takara Bio	
Cellartis^®^ Human iPS Cell Line 22	M	32	Human skin fibroblasts	Takara Bio	
GM25430	M	62	Human skin fibroblasts	Coriell	Old
AG28269	M	69	Human skin fibroblasts	Coriell	
AG27602	M	72	Human skin fibroblasts	Coriell	

## Data Availability

The data presented in this study are available on request.

## Supplementary Materials

The following supporting information can be downloaded at: https://www.mdpi.com/article/10.3390/antiox15060689/s1, Figure S1: Morphological progression during neural induction of aged human iPSCs into neural stem cells (NSCs); Figure S2: Characterization of iPSC-derived NSCs by Nestin/Sox2 immunostaining; Figure S3: Characterization of aged iPSC-derived neurons by MAP2 immunostaining; Figure S4: Characterization of aged iPSC-derived astrocytes by immunostaining for astrocytic markers under Geltrex-coated and uncoated conditions. (A) Characterization of differentiated astrocytes was performed using the astrocytic markers GFAP, S100B, and GLAST with corresponding merged images and DAPI staining, confirming the astrocytic phenotype of the differentiated cells (upper panels). (B) Representative images showing GFAP immunostaining with DAPI nuclear staining in aged iPSC-derived astrocytes cultured under coated and uncoated conditions (lower panels). Scale bars = 50 µm; Figure S5: Radar plot summary of age-associated mitochondrial alterations in iPSCs and derived neural cells; Figure S6. Mitochondrial and metabolic alterations in aged human iPSC-derived astrocytes compared to control astrocytes; Figure S7. Relative GBE-induced changes in mitochondrial respiratory parameters in young and aged iPSCs. Relative changes were calculated from group means for each respiratory parameter as percentage change versus the respective untreated control [(GBE−CTRL)/CTRL] × 100, allowing comparison of the relative magnitude of GBE responses between young and aged iPSCs despite differences in absolute scales; Figure S8. Relative GBE-induced changes in mitochondrial parameters in iPSC-derived neurons and astrocytes. Relative changes were calculated from group means as percentage change versus the respective untreated control [(GBE−CTRL)/CTRL] × 100, providing a descriptive normalization that allows comparison of the relative magnitude and direction of GBE responses across cell types.

## Abbreviations

ATP	Adenosine Triphosphate
BDNF	Brain-Derived Neurotrophic Factor
BMP-4	Bone Morphogenetic Protein 4
DHR	Dihydrorhodamine 123
DMSO	Dimethyl Sulfoxide
ECAR	Extracellular Acidification Rate
ETC	Electron Transport Chain
FCCP	Carbonyl cyanide-p-trifluoromethoxyphenylhydrazone
FGF	Fibroblast Growth Factor
GFAP	Glial Fibrillary Acidic Protein
GBE	Ginkgo biloba Extract
HBSS	Hanks’ Balanced Salt Solution
iPSC	Induced Pluripotent Stem Cell
MAP2	Microtubule-Associated Protein 2
MMP	Mitochondrial Membrane Potential
mtROS	Mitochondrial Reactive Oxygen Species
NSC	Neural Stem Cell
OCR	Oxygen Consumption Rate
PBS	Phosphate-Buffered Saline
ROS	Reactive Oxygen Species
ROCK	Rho-Associated Protein Kinase
TMRM	Tetramethylrhodamine Methyl Ester
