# Supplementary material for: Ginkgo Biloba Extract Ameliorates Age-Related Mitochondrial Deficits in Human iPSCs and Their Derived Neurons and Astrocytes
Source: Antioxidants (Basel). 2026 May 29;15(6):689. doi: 10.3390/antiox15060689 (PMC13296193; doi:10.3390/antiox15060689)
Supplement: Supplementary file 1 [file antioxidants-15-00689-s001.zip › antioxidants-4259153-supplementary.pdf]

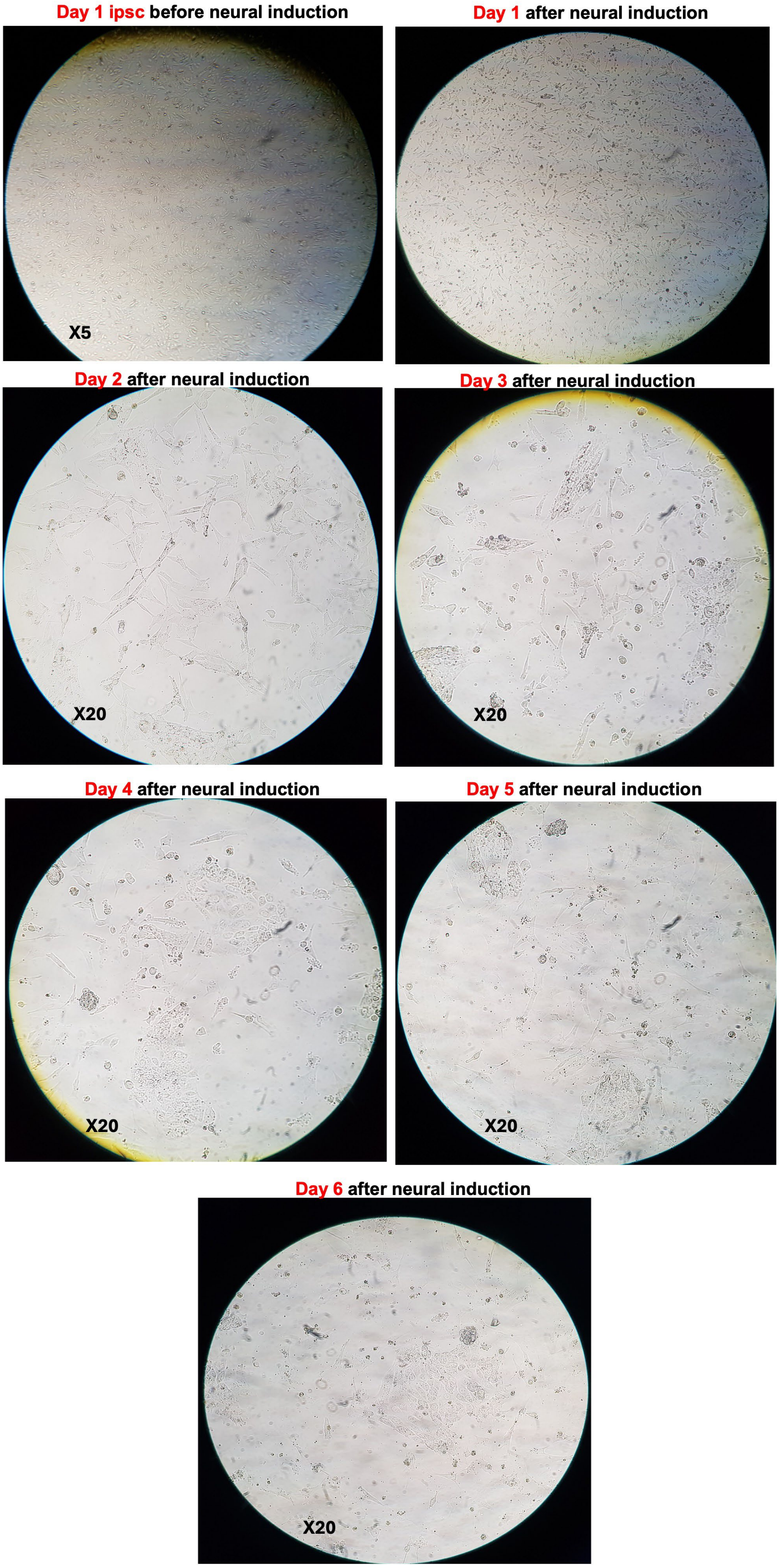

Figure S1: Morphological progression during neural induction of aged human iPSCs into neural stem cells (NSCs).

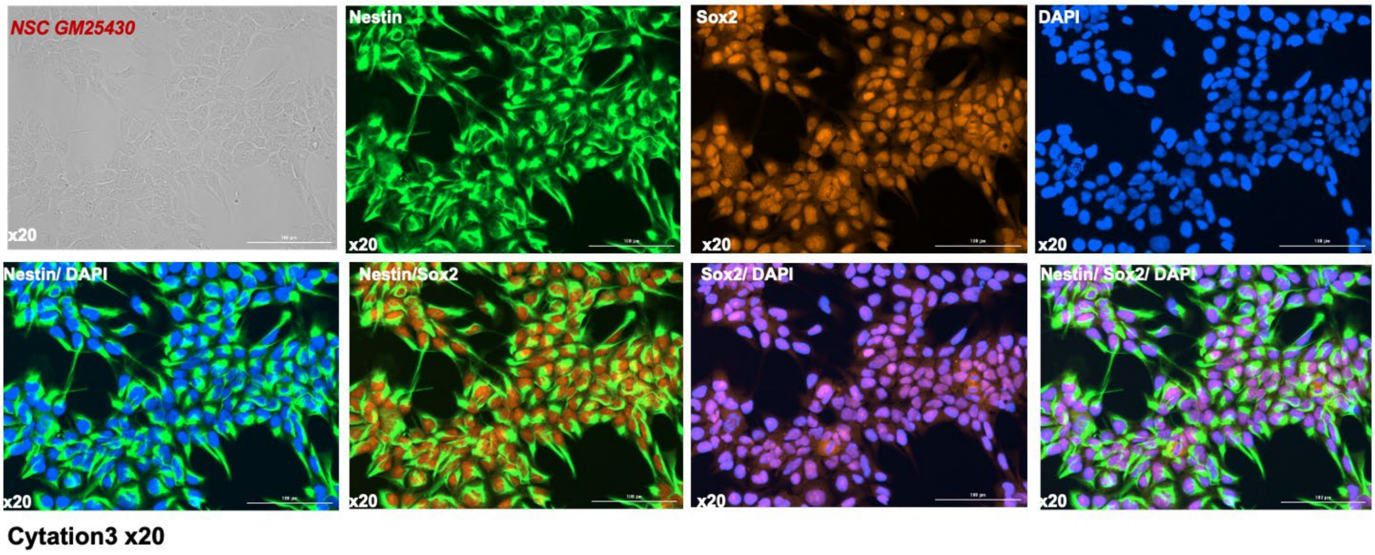

Figure S2: Characterization of iPSC-derived NSCs by Nestin/Sox2 immunostaining

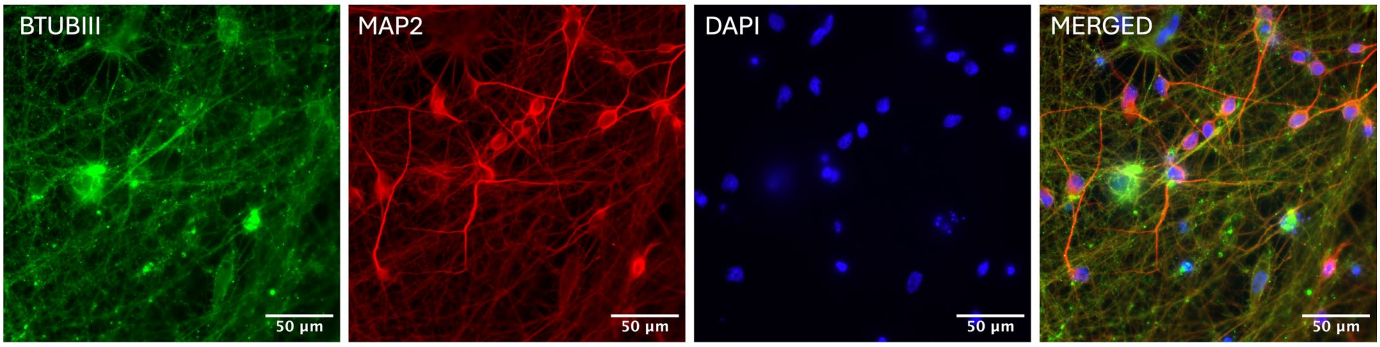

Figure S3: Characterization of aged iPSC-derived neurons by MAP2 immunostaining

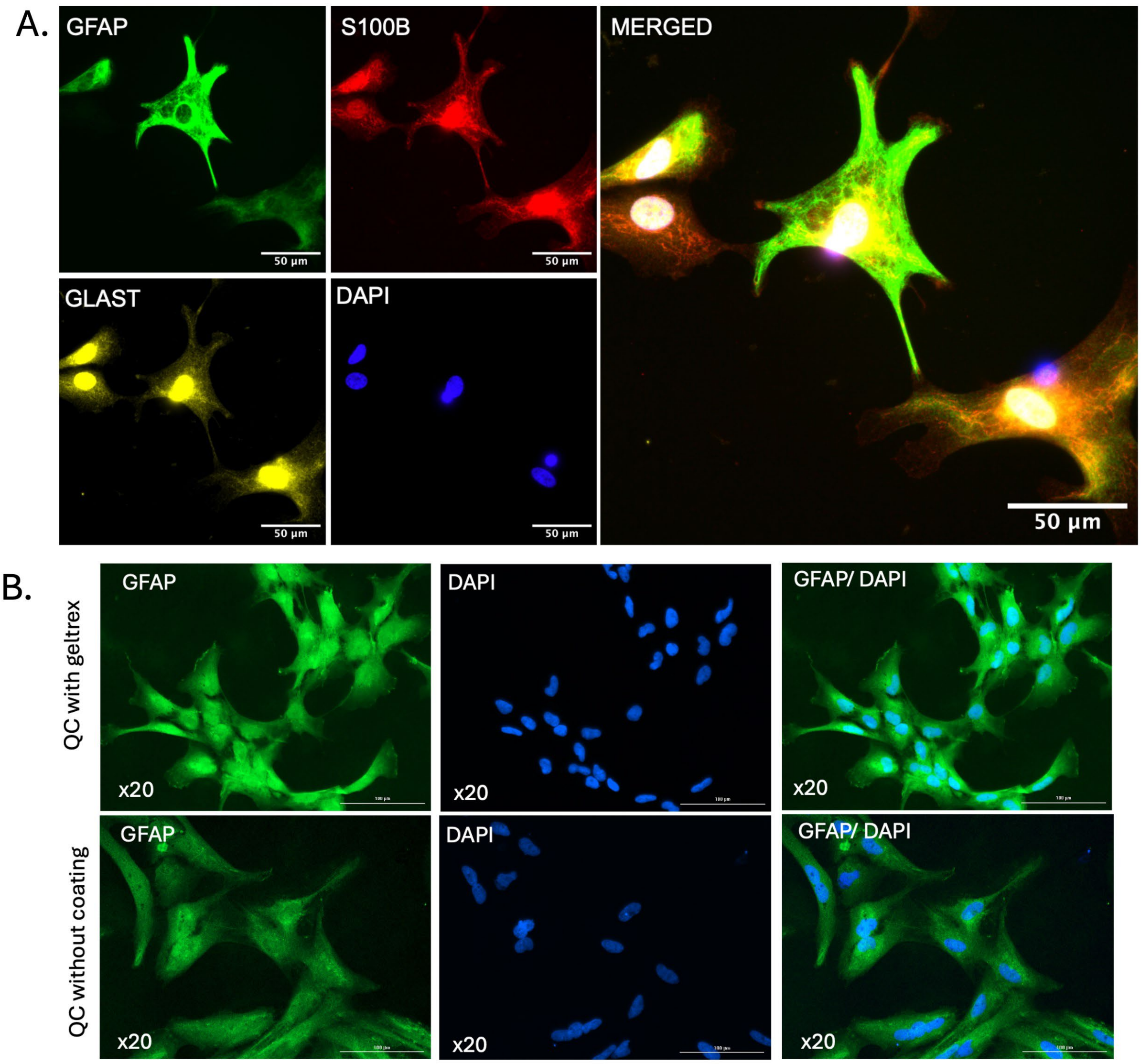

Figure S4: Characterization of aged iPSC-derived astrocytes by immunostaining for astrocytic markers under Geltrex-coated and uncoated conditions. (A) Characterization of differentiated astrocytes was performed using the astrocytic markers GFAP, S100B, and GLAST with corre-sponding merged images and DAPI staining, confirming the astrocytic phenotype of the differen-tiated cells (upper panels). (B) Representative images showing GFAP immunostaining with DAPI nuclear staining in aged iPSC-derived astrocytes cultured under coated and uncoated conditions (lower panels). Scale bars = 50 µm.

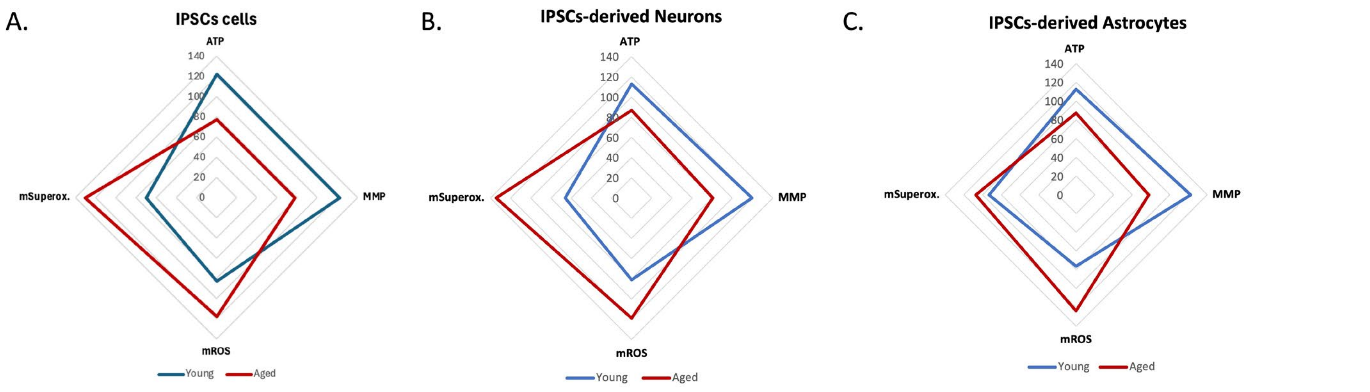

Figure S5: Radar plot summary of age-associated mitochondrial alterations in iPSCs and derived neural cells.

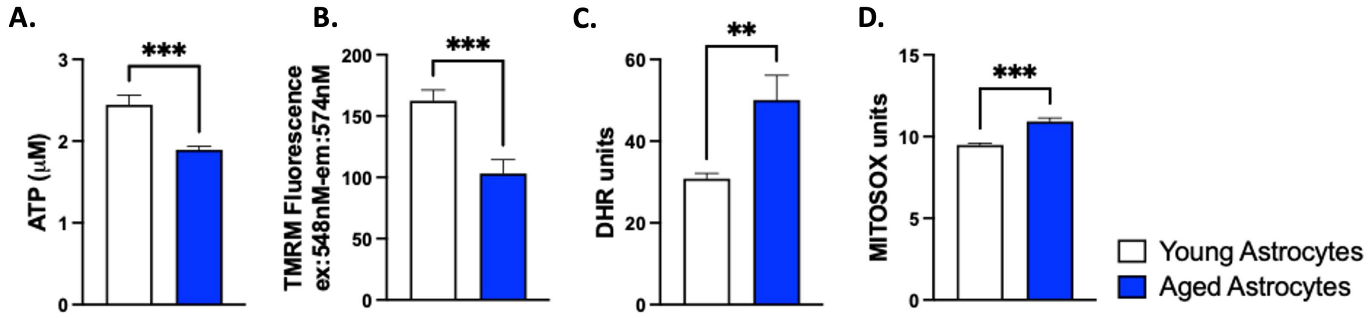

Figure S6. Mitochondrial and metabolic alterations in aged human iPSC-derived astrocytes compared to control astrocytes.

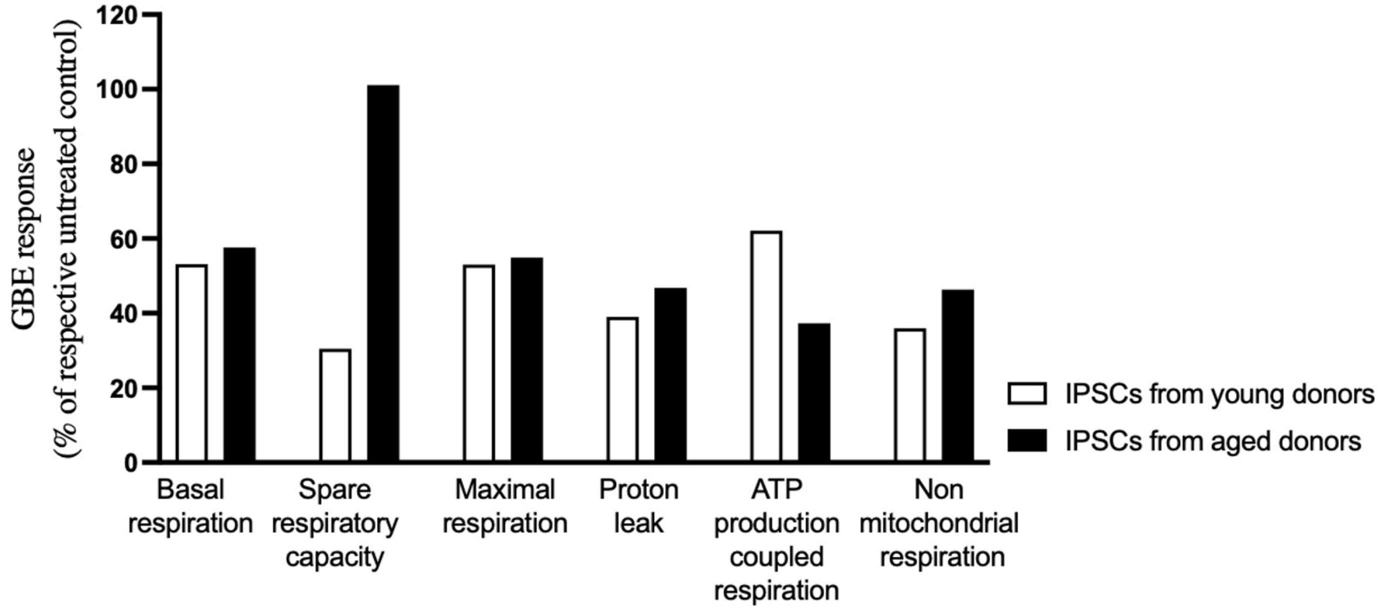

Figure S7. Relative GBE-induced changes in mitochondrial respiratory parameters in young and aged iPSCs. Relative changes were calculated from group means for each respiratory parameter as percentage change versus the respective untreated control [(GBE−CTRL)/CTRL] ×100, allowing

comparison of the relative magnitude of GBE responses between young and aged iPSCs despite differences in absolute scales.

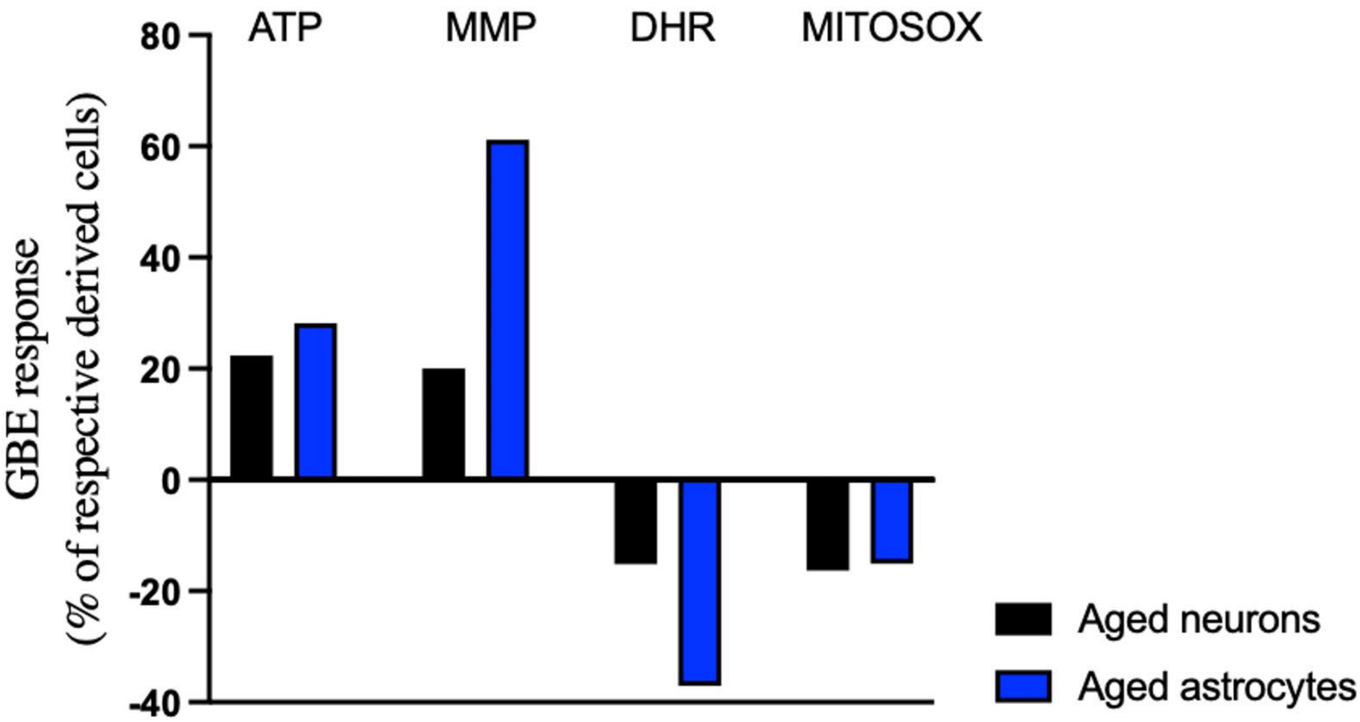

Figure S8. Relative GBE-induced changes in mitochondrial parameters in iPSC-derived neurons and astrocytes. Relative changes were calculated from group means as percentage change versus the respective untreated control [(GBE−CTRL)/CTRL] ×100, providing a descriptive normalization that allows comparison of the relative magnitude and direction of GBE responses across cell types.
